# Exploring the Bottom-Up Growth of Anisotropic Gold Nanoparticles from Substrate-Bound Seeds in Microfluidic Reactors

**DOI:** 10.1021/acsanm.3c00440

**Published:** 2023-04-07

**Authors:** Gail A. Vinnacombe-Willson, Joy K. Lee, Naihao Chiang, Leonardo Scarabelli, Shouzheng Yue, Ruth Foley, Isaura Frost, Paul S. Weiss, Steven J. Jonas

**Affiliations:** † Department of Chemistry and Biochemistry, University of California, Los Angeles, Los Angeles, California 90095, United States; ‡ Department of Pediatrics, University of California, Los Angeles, Los Angeles, California 90095, United States; § Department of Chemistry, University of Houston, Houston, Texas 77004, United States; ∥ Institute of Materials Science of Barcelona, ICMAB-CSIC, Campus UAB, Bellaterra 08193 Spain; ⊥ Department of Bioengineering, University of California, Los Angeles, Los Angeles, California 90095, United States; # Department of Materials Science and Engineering, University of California, Los Angeles, Los Angeles, California 90095, United States; ¶ California NanoSystems Institute, University of California, Los Angeles, Los Angeles, California 90095, United States; ● Eli & Edythe Broad Center of Regenerative Medicine and Stem Cell Research, University of California, Los Angeles, Los Angeles, California 90095, United States

**Keywords:** gold nanostars, microfluidic devices, substrate growth, seed-mediated growth, plasmonic nanoparticles, surface-enhanced Raman scattering, thermoplasmonics

## Abstract

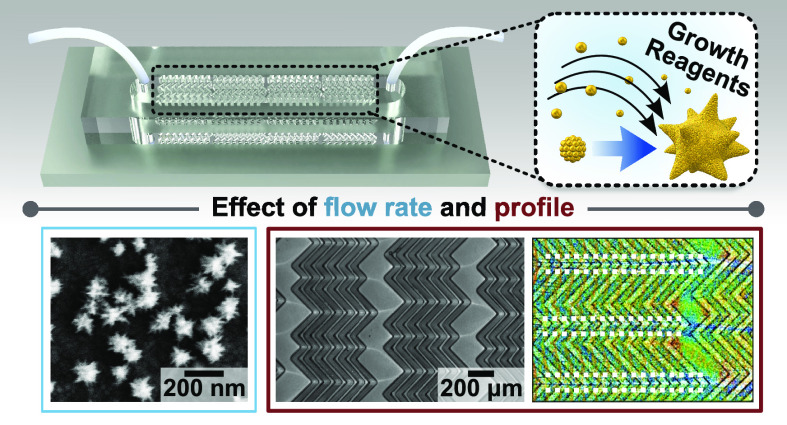

We developed an unconventional seed-mediated *in situ* synthetic method, whereby gold nanostars are formed directly on the internal walls of microfluidic reactors. The dense plasmonic substrate coatings were grown in microfluidic channels with different geometries to elucidate the impacts of flow rate and profile on reagent consumption, product morphology, and density. Nanostar growth was found to occur in the flow-limited regime and our results highlight the possibility of creating shape gradients or incorporating multiple morphologies in the same microreactor, which is challenging to achieve with traditional self-assembly. The plasmonic–microfluidic platforms developed herein have implications for a broad range of applications, including cell culture/sorting, catalysis, sensing, and drug/gene delivery.

Plasmonic nanostructures are key elements for on-chip platforms that require light-triggered heating,^1,2^ hot electron catalysis,^3,4^ and enhanced localized electromagnetic fields,^5,6^ which are useful properties for applications as cancer diagnostic tools,^7^ chemical and biological sensors,^5,6^ anticounterfeiting,^8^ and microreactors.^3,4^ There is great interest in batch fabrication of plasmonic nanoparticles in which precise control over nanoparticle composition, morphology, and surface chemistry is facilitated through bottom-up chemistry.^9^ Chemical synthesis provides unmatched control over the particles’ properties, enabling selection of the desired reactivity and plasmonic response for the intended applications.^9^ General strategies that leverage bottom-up chemical synthesis for incorporating plasmonic nanoparticles in on-chip systems include: (*i*) self-assembly and patterning with premade colloidal nanoparticles^10−12^ and (*ii*) *in situ* growth, where the particles are instead grown directly on the substrate.^13^ The latter approach has the potential to simplify fabrication processes for plasmonic–microfluidic platforms, avoiding multistep batch synthesis and time-consuming ligand exchange.^10,13,14^ Furthermore, *in situ* growth can improve the density of the anisotropic nanoparticle coatings.^1,14^ However, until now, the synthetic mechanisms of direct *in situ* growth of plasmonic nanoparticles on the internal walls of microreactors have remained largely unexplored, especially using flow profiles beyond standard laminar flows.^1,3,4^ Recently, *in situ* microfluidic growth was performed in glass capillaries.^1^ However, since glass microchannels are nonconductive, obtaining detailed morphological characterization with scanning electron microscopy (SEM) requires the addition of a thin conductive coating or the use of environmental SEM. Moreover, advanced sample processing techniques such as ultramicrotomy or focused ion beam (FIB) slicing would be required for transmission electron microscopy (TEM) characterization. In addition, glass capillaries lack versatility for testing the effects of different flow profiles on the growth of the nanostructures beyond laminar flows, also limiting the types of applications that can be targeted (i.e., tunable flow profiles are utilized in particle and cell isolation/sorting;^7^ microreactors with more complex geometries can be engineered to combine multiple solutions or reagents selectively). Overall, these barriers limit the synthetic interrogation of *in situ* anisotropic nanoparticle growth under varied flow environments, which is a key aspect to investigate to improve the development of plasmonic microchannels.^7,15−17^

Here, we interrogate the *in situ* anisotropic synthesis of gold nanoparticles within microreactors in both laminar and chaotic flow environments. We opted to test our unconventional *in situ* synthetic approach in polydimethylsiloxane (PDMS) “herringbone” (HB) chaotic mixers with 3D staggered features, which create chaotic flows useful for particle/cell sorting and can be applied as microreactors due to their ability to promote reagent/heat exchange.^7^ Our device fabrication procedure begins by binding the PDMS channel to either glass or indium tin oxide (ITO)-coated glass using (3-aminopropyl)triethoxysilane (APTES) as an adhesion layer (Figures 1AI,II and S1). The resulting PDMS-microfluidic channels are shown in the insets of Figure 1C,D. Details on the HB design are shown in Figure 1B and in the Supporting Information. Next, we applied *in situ* overgrowth to prepare dense branched gold nanostar (AuNST) coatings to test the effect of different flow environments on anisotropic growth. The AuNSTs are advantageous for an extensive range of on-chip applications since they exhibit significant electromagnetic field enhancements at their sharp tips,^1,18^ high extinction within the near-infrared biological transparency window,^1^ capabilities to efficiently convert light to heat,^19,20^ biocompatibility,^20^ and high surface areas.^21^ Altogether, compared to colloidal batch protocols, performing seed overgrowth *in situ* (rather than tethering presynthesized anisotropic products) has been reported to improve product density and to limit the formation of byproducts that result from secondary nucleation.^1,14^ The growth of AuNSTs was directed to occur specifically on the APTES-coated channel walls via substrate functionalization with colloidally prepared gold seeds (Figure 1AIII). Seeding conditions were kept constant for all experiments: colloidally prepared seeds were functionalized onto the channel walls at a constant flow rate (50 μL/min) for 60 min. After the seeded channels were rinsed with 18.2 MΩ (ultrapure grade) water, a nanoparticle growth solution, containing gold salt, capping ligand laurylsulfobetaine, and weak reductant ascorbic acid, was introduced at the selected flow rate over a fixed 3 min growth time (Figure 1AIV; see Supporting Information for experimental details). Due to the hydrophobicity of PDMS, a key step for flow-mediated synthesis in microchannels with complex 3D features involves exposing the assembled devices to air plasma treatment to assist in wetting (Figures S2 and S3). SEM evaluation of the channels revealed the successful *in situ* formation of AuNSTs within both the featureless and HB microreactors (Figure 1C,D). Furthermore, SEM characterization showed that the nanostructures retain their morphologies, even after 1 month (Figure S4). Environmental SEM characterization also confirmed the presence of branched nanostructures on the PDMS (Figure S5). The resulting AuNSTs feature one major highly branched, high aspect ratio product, which has characteristics that provide the highest efficiency for light-to-heat conversion and sensitivity for bioimaging and sensing.^1,22^

**Figure 1:**
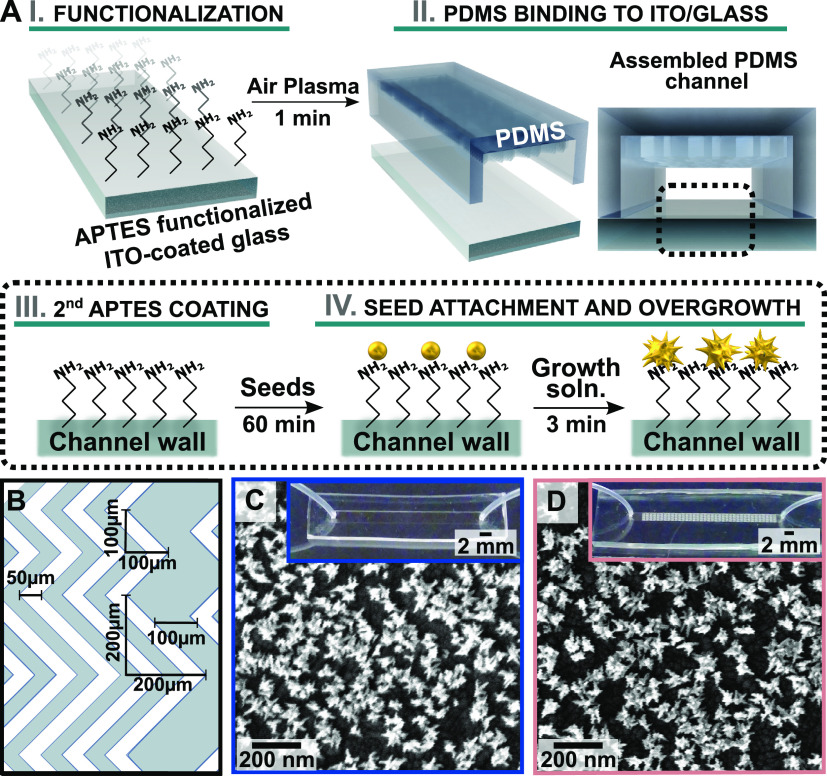
(A) Schematic showing (I) functionalization of indium tin oxide (ITO)-coated glass with (3-aminopropyl)triethoxysilane (APTES) adhesion layer to facilitate binding of a polydimethylsiloxane (PDMS) channel for assembly of the microfluidic devices (II). After channel fabrication, (III) a second APTES coating was applied to provide amine moieties for (IV) anchoring gold colloidal seeds to the growth surface and directing overgrowth at the substrate to produce anisotropic gold nanostar structures when a growth solution containing gold, ascorbic acid, and shape-directing reagents was flowed into the channels. (B) Diagram of the herringbone (HB) structure. White represents raised areas of the PDMS (*h* = 50 μm, total channel height = 90 μm). (C,D) Scanning electron microscopy images of nanostars on ITO synthesized in (C) featureless and (D) HB channels. Insets: Digital photographs of the microfluidic chips.

In prior work, it was shown that increasing the total volume of reagents passed through the channel (i.e., by increasing the growth time at a fixed flow rate) intuitively leads to increased growth of the nanostructures.^1^ Here, we sought to interrogate further the effect of flow rate by applying a fixed total volume of growth solution (and therefore fixed quantity of reagents) under different flow rates and corresponding growth times (42 μL/min for 9 min, 63 μL/min for 6 min, 125 μL/min for 3 min, and 375 μL/min for 1 min; for a total of ∼375 μL). Surprisingly, it was found that although the same quantity of precursors was introduced into the channels in all cases, each condition produced different products, as confirmed via visual inspection and SEM characterization (Figure S6). The products grown at 125 μL/min for 3 min had the most branching, while the products at the lower flow rates (42 μL/min and 63 μL/min) produced nanoparticles with fewer sharp branches, indicating that slower delivery of the reagents to the substrate may disfavor the formation of kinetically favored high aspect ratio AuNSTs. When growth was performed at 375 μL/min for 1 min, the resulting AuNSTs appeared slightly undergrown (less branching, smaller) compared to those grown at 125 μL/min for 3 min, indicating that the total growth volume at this flow rate was likely insufficient for producing highly branched products. In sum, performing the synthesis with fixed reagent volumes produced dissimilar products. Thus, we show that while both total reagent volume and flow rate both can affect the growth, the flow rate in particular has a predominant influence on the production of AuNSTs.

Nanostar growth in flow was also observed to produce a color gradient proceeding from the inlet to the outlet, evident by eye and by conventional ultraviolet (UV)–visible spectroscopy (Figures S7 and S8). Spectroscopic characterization showed that the intensity of the gradients, evaluated using the difference in extinction at 400 nm between the inlet and outlet regions of the channels was more drastic at low flow rates (42 μL/min, 9 min; 63 μL/min, 6 min) compared to the channels prepared at higher flow rates (125 μL/min, 3 min; 375 μL/min, 1 min) (Figure 2). After establishing that the flow rate has a dominant effect compared to growth volume, we next tested the AuNST growth under fixed growth times (3 min), using different flow rates. Ultimately, the gradients could either be caused by variations in product morphology or density. Easily tunable gradients resulting from products with differing morphologies are of greater interest, considering that recently substrates with plasmonic shape gradients incorporating nanostructures with gradually increasing branching were applied as “plasmonic libraries” for rapidly screening surface-enhanced Raman scattering (SERS) efficiencies.^22^ In this work, we fabricate similar gradual AuNST shape gradients without the need for lengthy self-assembly (∼4 h total fabrication time including substrate construction, ∼2.5 h, and synthesis, ∼1.5 h).

Altogether, while visual inspection and conventional UV–visible spectroscopy showed changes in intensity at the distinct regions similar to those of the previous experiments (Figure 2A,B and Figure S8), little information could be gleaned regarding changes in the nanoparticle morphology due to the broad localized surface plasmon resonance (LSPR) associated with branched structures and the low intensity/optical interference caused by the substrate. Thus, the capability to bind the PDMS microchannels to a conductive ITO base reliably was a key development, enabling morphological characterization via high vacuum electron microscopy, which was used to evaluate various parameters that were useful for comparing the products at different growth conditions (see Supporting Information for details of SEM sample preparation). We assessed (1) product morphology; (2) AuNST density, defined as number of nanoparticles/μm^2^; and (3) percentage of gold coverage on the substrate surface, defined as the total area of the substrate covered by gold (a parameter that is affected both by the density of the nanostructures and their size), which was determined by calculating the percentage of white pixels in SEM images following binarization with MATLAB software (Supporting Information section 6). Furthermore, the implementation of a conductive substrate maybe useful for electrochemical sensors or interrogating current-responsive cell cultures, for instance.^23−25^ The SEM images showed that less-developed AuNSTs were present at the outlets, with the underlying substrate being more visible (with a lower percentage of gold covering the surface) compared with the inlet (Figures 2C–E and S9). The differences in gold coverage between the inlet and outlet were used to estimate the extent of the gradient (Figure 2F). It was found that the total gold coverage of the substrate decreased by ∼20% for both channel geometries, while the density remained consistent between the two regions (Figure S10). These analyses support that the observed macroscale color gradation was predominantly caused by morphological differences of the products along the channel (rather than differences in density).

**Figure 2:**
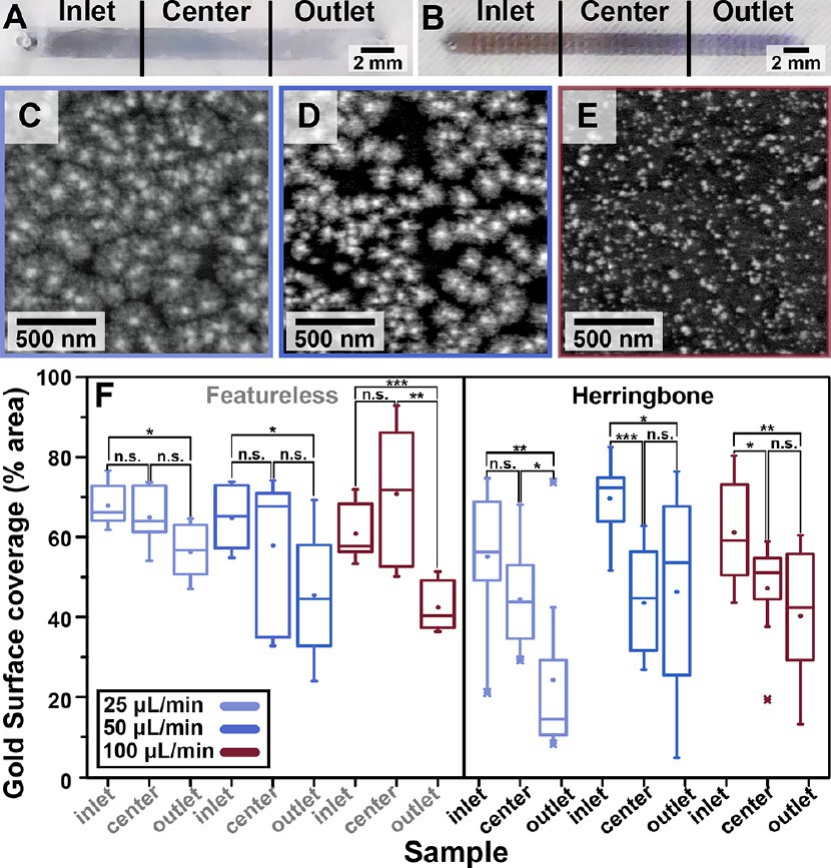
(A,B) Digital photographs of (A) featureless and (B) herringbone (HB) channel gradients. (C–E) Scanning electron microscopy images of gold nanostars grown at a flow rate of 100 μL/min. Images correspond to the (C) inlet, (D) center, and (E) outlet of a channel. (F) Percentage of the surface covered by gold between the inlet, center, and outlet regions showing products grown in featureless (left) and HB channels (right) at different flow rates.

We attribute the shape gradient to the gradual consumption of reagents as the solution flows through the device. As there is no significant vertical mass transfer in the laminar flow, the reagents near the surface are depleted. In the HB channels, although there is vertical mixing to replenish reagents at the surface, the gradient could be caused by a reduction of the total solution concentration. This observation is consistent with the reaction occurring in the flow-limited regime, where the rate-determining step of AuNST overgrowth is the rate of the gold precursor reaching the seeds, which is dictated by the flow rate. In other words, the seeds at the outlet receive less gold, limiting the formation of AuNSTs, which are kinetically favored.^26^ Ultimately, this result highlights the possibility of utilizing flow to synthesize nanoparticles with different morphologies side-by-side on the same substrate, which is prohibitively challenging to perform using self-assembly based methods.^22^ We find that products within ∼1 cm sections of the 2 mm-wide channels appear relatively uniform. Therefore, if a substrate with uniform nanostructures is required, the channel length should be kept at or below 1 cm, and the channel height and/or width should be increased if larger channel volumes are needed for the desired application.

The HB micromixer geometry provides a platform to test the hypothesis of flow-limited growth further since the deviation from laminar flow in different areas of the HB could enable the creation of different growth patterns. Examining the HB channel, the intensity of the coloration caused by the AuNSTs appears to follow a pattern laterally across the channel with low-intensity areas located at certain “peaks” of the HB features (Figures 3A and S11). Furthermore, when the flow direction is reversed, the lateral pattern changes (Figure 3B). These observations are supported by examining simulated trajectories, which showed that the growth solution is directed away from the peaks (indicated by dotted lines in Figure 3, COMSOL Multiphysics software). These data further support the occurrence of flow-limited growth within the microchannels and show that different growth patterns can be accessed by tuning the micromixer geometry.

**Figure 3:**
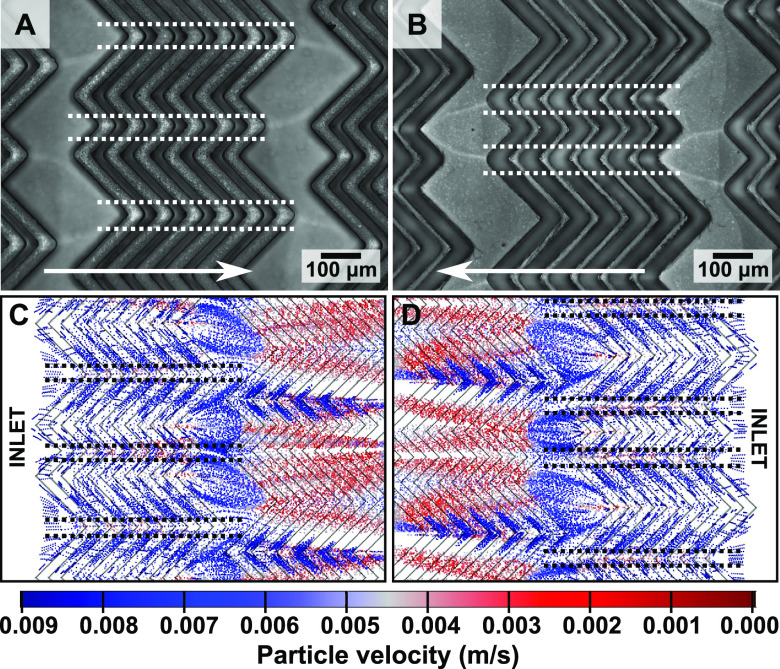
(A,B) Bright-field microscope images of the center of the herringbone (HB) channels when applying flow (A) to the right and (B) to the left (indicated by the white arrows). (C,D) Top view of the simulated flow trajectories showing greater concentrations of the 10 nm contrast particles away from the HB peaks (indicated by dashed lines). The colors represent the flow velocities of the contrast particles. More images of the channels and simulated flows can be found in the Supporting Information, Figures S11 and S12 and Videos S1 and S2).

Despite the indicators that growth is primarily flow-limited, we noticed a counterintuitive result where, in Figure 2F, there does not appear to be a significant difference in the gold coverage at the center of the channels at flow rates between 25 and 100 μL/min after growth at a fixed time of 3 min. However, if the growth is truly flow-limited, then one would expect the products to change when different flow rates are applied. To investigate this question further, we examined AuNSTs synthesized under a broader range of flow rates, up to 1000 μL/min (Figure 4A,B), comparing the product density, size, and percentage of surface gold coverage as described earlier (Figures S9 and S15). For featureless channels, up to ∼500 μL/min the size (∼100 nm), density (∼60 AuNSTs/μm^2^), and percentage of gold coverage (∼50%) continue to remain comparable. Note, regarding nanoparticle density, we make the assumption that the density of the seeds is either equivalent or proportional to the density of the final products, since the limit of resolution of the SEM instrument does not permit reliable imaging of particles much smaller than 5 nm (seeds ∼2 nm). Furthermore, we designed our fabrication scheme to address this technical limitation and this assumption is reasonable due to the slow seeding flow rate and long incubation times. However, when high flow rates (>500 μL/min) are applied, the structures overgrow to the point of almost completely covering the underlying substrate and lose the characteristic sharp branches of the AuNSTs (Figure 4A and Figures S13 and S15). In the HB channel, a similar trend is observed where the average size (∼60 nm), density (∼100 AuNSTs/μm^2^), and gold coverage (∼50%) remain relatively unchanged until partial gold films and overcrowded structures are produced as flow rates exceed 250 μL/min (Figure 4B,C and Figures S14 and S15). Thus, we found that HB channels reach significantly higher gold coverages on the surface at lower flow rates than featureless channels, indicating that vertical mixing leads to more efficient consumption of the growth reagents.

**Figure 4:**
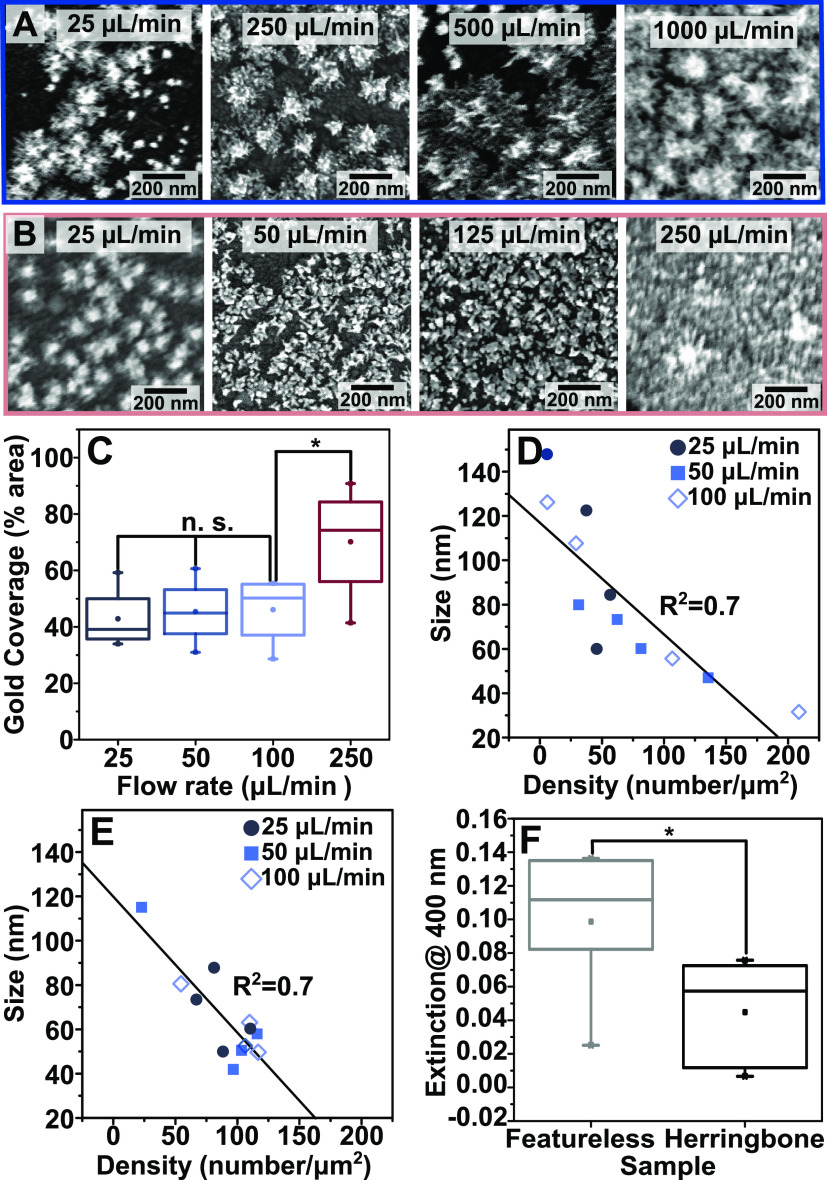
(A,B) Scanning electron microscopy (SEM) images of gold nanostars grown at the specified flow rates over 3 min in (row A) featureless and (row B) herringbone (HB) channels. (C) Gold coverage of the surface at different flow rates in the HB channels. (D,E) Relationship between average nanoparticle density and size in (D) featureless and (E) HB channels (each point represents one sample). (F) Extinction at 400 nm of the outlet solutions collected from the different devices aged for 2 h. Additional analyses are provided in Figures S15 and S16, and more SEM images are provided in Figures S17 and S18.

While the products synthesized between 25 and 100 μL/min in both channel configurations are not significantly different, contrary to what is expected for flow-limited growth, this result can be rationalized by the dominant influence of product density, which is dictated by the APTES coating and seed attachment yields.^1^ With both channel geometries, there is a negative correlation between AuNST size and density at all applied flow rates (correlation coefficients of −0.80 and −0.83 for HB and featureless channels, respectively). Thus, attaining high reproducibility in seed surface functionalization for *in situ* growth of kinetically favored products is critical for future detailed mechanistic studies. The result in Figure 4F suggests that another advantage of the HB geometry is that the chaotic mixing in the channel enables more uniform and reproducible coverage for both APTES and seeds, which ultimately translates to enhanced control over product size and density.

Lastly, UV–visible spectroscopic analyses of the growth solution exiting the microfluidic channel were performed to evaluate the efficiency of reagent consumption in the channels quantitatively (Figures 4F and S16). It is established that nanoparticles can spontaneously nucleate in the growth solution even without the addition of seeds, especially for the synthesis of kinetic products like AuNSTs.^27−29^ The spontaneous reduction of the gold precursor in the solution causes it to change color from clear to blue/purple, and we applied UV–visible spectroscopy to monitor this process. We found that significant secondary nucleation begins after ∼10–20 min for the synthetic parameters used here (Figure S16). Next, we utilized this so-called “homogeneous” or “secondary” nucleation to determine the quantity of gold remaining in the growth solution as it exits the channel. The absorbance at 400 nm is dominated by interband transitions in metallic gold. So, the intensity of the absorbance at 400 nm can be used to compare the final gold atom concentrations in the growth solution collected at the outlets of both types of devices.^30^ From our spectroscopic measurements, the reduction of the gold precursor in the growth solution at the outlet is complete after 2 h (extinction at 400 nm no longer changes, Figure S16). Comparing the 2 h aged outlet solutions at 250 μL/min, the HB samples had roughly half the extinction of the featureless channels (Figure 4F), indicating that gold precursor consumption is *ca*. twice as efficient.

In summary, the successful growth of AuNSTs in HB channels demonstrates that our *in situ* fabrication method can be used to create dense coatings of morphology-controlled nanostructures on complex substrates, including microchannels with 3D features that can be applied for biomedical applications such as cell sorting^1^ and theranostics.^2^ To the best of our knowledge, this work provides the first demonstration that the flow profile can be applied to alter the growth kinetics of plasmonic nanoparticles growing directly on a substrate. Furthermore, the identification that the synthesis of AuNSTs occurs in the flow-limited regime informs the development of similar *in situ* flow growth schemes targeting kinetic nanoparticle products. Our initial results indicate that more detailed studies interrogating the size and density relationship could provide additional mechanistic insights into substrate seed-mediated growth. Overall, our results establish that the flow conditions, reaction kinetics, and seeding yields are all important factors affecting the product shape and density. In the future, beyond standard and environmental SEM, high-resolution electron microscopy characterization using FIB/ultramicrotomy along with TEM, for example, could assist in expanding fundamental synthetic investigations on *in situ* overgrowth via crystallographic characterization. Additionally, UV–visible–NIR microscopy could also be used to evaluate the LSPR peak position and intensity. Ultimately, we lay the groundwork for the straightforward fabrication of complex thermoplasmonic nanoparticle-integrated microfluidic systems that incorporate chaotic flows, which can be broadly applied for cell isolation/sorting, chemical/biological sensing, drug delivery/screening, and catalysis.

## Supporting Information Available

Materials and methods, including materials, preparation of polydimethylsiloxane channels, microfluidic synthesis, and gold nanostar characterization; additional details on polydimethylsiloxane channel binding process to indium tin oxide; environmental SEM characterization; MATLAB evaluation of the percentage of the surface covered with gold; additional data and numerical simulations; additional electron microscopy characterization of gold nanostars (PDF)
Simulated flows as described in Figure 3 (Video S1, MP4; Video S2, MP4)
