# Supplementary material for: Exploring the Bottom-Up Growth of Anisotropic Gold Nanoparticles from Substrate-Bound Seeds in Microfluidic Reactors
Source: ACS Appl Nano Mater. 2023 Apr 7;6(8):6454–60. doi: 10.1021/acsanm.3c00440 (PMC10152454; doi:10.1021/acsanm.3c00440)
Supplement: Supplementary file 1 — an3c00440_si_001.pdf [file an3c00440_si_001.pdf]

Supporting Information

Exploring the Bottom-Up Growth of Anisotropic Gold Nanoparticles from Substrate-Bound Seeds in Microfluidic Reactors

Gail A. Vinnacombe-Willson,[a*†] Joy K. Lee,[b†] Naihao Chiang,[c] Leonardo Scarabelli,[d] Shouzheng Yue,[e] Ruth Foley,[e] Isaura Frost,[e] Paul S. Weiss,[a,d,f,g]* & Steven J. Jonas[b,g,h]*

a. Department of Chemistry and Biochemistry, University of California, Los Angeles, Los Angeles, California 90095, United States
b. Department of Pediatrics, University of California, Los Angeles, Los Angeles, California 90095, United States
c. Department of Chemistry, University of Houston, Houston, Texas 77004, United States
d. Institute of Materials Science of Barcelona, ICMAB-CSIC, Campus UAB, Bellaterra, 08193 Spain
e. Department of Bioengineering, University of California, Los Angeles, Los Angeles, California 90095, United States
f. Department of Materials Science and Engineering, University of California, Los Angeles, Los Angeles, California 90095, United States
g. California NanoSystems Institute, University of California, Los Angeles, Los Angeles, California 90095, United States
h. Eli & Edythe Broad Center of Regenerative Medicine and Stem Cell Research, University of California, Los Angeles, Los Angeles, California 90095, United States
**To whom correspondence should be addressed.*
[†]Equal contribution

Contact information for corresponding authors:
G. A. Vinnacombe-Willson: gvinnacombe@cicbiomagune.es
P. S. Weiss: psw@cnsi.ucla.edu
S. J. Jonas: sjonas@ucla.edu

CONTENTS:

1. Materials and Methods

1.1. Materials

1.2. Preparation of polydimethylsiloxane channels

1.3. Microfluidic synthesis

1.4. Gold nanostar characterization

2. Additional Details on Polydimethylsiloxane Channel Binding Process to Indium Tin Oxide

3. Environmental Scanning Electron Microscopy Characterization

4. Conventional Ultraviolet-Visible Spectroscopy Characterization

5. MATLAB Evaluation of the Percentage of the Surface Covered with Gold

6. Additional Data and Numerical Simulations

7. Additional Electron Microscopy Characterization of Gold Nanostars

1. Materials and Methods

1.1. Materials

All analytical grade reagents, silver nitrate ($AgNO_3$; 99%, CAS: 7761-88-8), $HAuCl_4 \cdot 3H_2O$ (99%, CAS: 16961-25-4) L-ascorbic acid (99%, CAS: 50-81-7), *N*-dodecyl-*N*,*N*-dimethyl-3-ammonio-1-propanesulfonate (LSB; 99%, CAS: D0431), hydrochloric acid (HCl; ACS Reagent 37%, CAS: 7647-01-0), nitric acid (HNO_3; 15.8 M, Fisher Scientific, CAS: 7697-37-2), (3aminopropyl)triethoxysilane (APTES, 99%, CAS: 919-30-2), cetyltimethylammonium chloride (CTAC; 25 wt % in H_2O, CAS: 112-02-7), and trichloro(*1H,1H,2H,2H*-perfluorooctyl)silane (97%, CAS: 78560-45-9) were purchased from Millipore Sigma. High performance liquid chromatography (HPLC)-grade sub-micron filtered water (CAS: 67-63-0) and 200 proof ethanol (CAS: 64-17-5) were obtained from Fisher Scientific. All glassware and magnetic stir bars used for synthesis were cleaned thoroughly with *aqua regia* (3:1 concentrated HCl to HNO_3) and rinsed with HPLC-grade water (Fisher Scientific) before use. *Warning: Aqua regia* solution is extremely corrosive and reactive. Care is needed in its preparation and usage. Polydimethylsiloxane (PDMS, Sylgard 184) was purchased from Dow Corning (Michigan, USA). Indium tin oxide (ITO) coated glass slides were purchased from MSE supplies (0.7 mm 7-10 Ohm/Sq ITO Coated Glass Substrate, 50 mm × 50 mm × 0.7 mm).

1.2. Preparation of polydimethylsiloxane channels

Prior to replica molding PDMS channels, photolithographically prepared silicon masters were pretreated with trichloro(*1H,1H,2H,2H*-perfluorooctyl)silane to serve as a release layer. Vapor-phase deposition of the trichloro(*1H,1H,2H,2H*-perfluorooctyl)silane onto the silicon master was performed as follows: 4 µL of the silane was placed in a reservoir in a vacuum

desiccator with the masters (placed vertically on the side walls) and was placed under vacuum for 20 min using a benchtop vacuum pump. The substrates were rinsed with isopropyl alcohol and completely dried with compressed air. The masters could be re-used ~5 times before the silane coating had to be reapplied.

The channels were prepared using soft lithography by thoroughly mixing PDMS at a standard 10:1 base to curing agent ratio by weight. After mixing, the solution was degassed by centrifuge at 3500 rpm for 2 min. Then, the PDMS mixture was poured onto the silicon master, degassed a second time for ~10 min, and then cured for 1 h at 80 °C.

The PDMS-glass channels were fabricated by simultaneously exposing both the glass substrate and the PDMS channel to 30 s – 1 min of air plasma (8 sccm, 100 W, Henniker Plasma HPT200). The channel and substrate were immediately placed in contact after plasma treatment, then placed in a 60 °C oven to facilitate binding for at least 1 h before use. Note that they can also be stored in the oven overnight. For ITO-coated glass, the substrates were functionalized with APTES before the plasma activation and binding process: the substrates were incubated in a 5% w/v APTES ethanolic solution at 60 °C for 5 min. The slides were then rinsed well with ethanol and dried with nitrogen. After the APTES functionalization, ITO and the PDMS channels were air plasma treated and bound in the same way as the PDMS-glass devices.

After the channels were successfully assembled, the devices (with inlet and outlet holes already punched) were placed in the air plasma cleaner for 1 min to increase the hydrophilicity of the internal channel walls. The channels were then pre-wetted with 200 proof ethanol at 250-500 µL/min for ~2 min. When herringbone channels were used, extra care was taken during this step to ensure that bubbles did not remain trapped in the three-dimensional (3D) features. Bubbles found within the channels were released by tapping the channel either by hand or with the back of

a pair of tweezers while the wetting ethanol solution was flowing through the channel at 250-500 μL/min. Then, a 5% w/v APTES ethanolic solution (for facilitating substrate binding of the colloidal seeds) was introduced and allowed to flow into the channel for 1 min at 250-500 μL/min. Then, the channels were quickly placed in a 60 °C oven for 8 min with tubing attached and liquid still inside. (*N.B.,* without the tubing the APTES solution will evaporate, if this appears to be happening, the tubing should be plugged with a syringe so that the solution is able to warm to 60 °C without evaporating). Immediately afterwards, the channels were rinsed with ethanol at 250-500 μL/min for 5 min.

1.3. Microfluidic synthesis

Colloidal seeds were fabricated by a previously established protocol,[1] where aqueous $NaBH_4$ (final concentration of 0.64 mM) was added rapidly to a solution containing 0.27 mM $HAuCl_4$ and 100 mM cetyltrimethylammonium chloride (CTAC) under vigorous stirring. The seed solution was flowed through the channels at 50 μL/min for 60 min using a syringe pump (Harvard, Chemyx 400). Here, it is again important for the herringbone channels that bubbles are not trapped in the 3D features, otherwise the growth will not be uniform. The devices were then gently hand-rinsed with water using a syringe. (*N.B.,* Other brands of syringe pumps were tested, and we observed that these alternatives would significantly heat up over a long period of use, negatively affecting the reproducibility of the synthesis. It is important to verify that the brand of pump used does not generate considerable heat during operation.) Lastly, the tubing at the inlet of the device is replaced following the seeding step with fresh/clean tubing to avoid growth within the tubing prior to introducing the growth solution to the channel.

Next, a 100 mM aqueous solution of ascorbic acid was prepared and quickly added (to a 5 mM final concentration) to a prepared solution containing gold salt (0.75 mM) and shape directing reagents HCl (10 mM), $AgNO_3$ (0.15 mM), and laurylsulfobetaine (100 mM). The ascorbic acid solution and growth solution must be quickly mixed immediately after addition so that the gold precursor can be uniformly reduced from Au^{3+} to Au^{1}, which is indicated by the change in color from pale yellow to clear. Once the solution turns clear (within a few seconds), the solution was immediately flowed into the seed-functionalized channel at the selected flow rate for 3 min (**Figure 1AIV,** Supporting Information).

NB- The growth solution must be used immediately; otherwise, secondary nucleation of colloidal stars will occur and these products will introduce competition with the selective growth on the substrate as reported in previous work.[S1] The growth solution has been tailored such that significant secondary nucleation will not occur for at least 10 min.

Growth was continued for 3 min after which the pump was stopped, and the channels were immediately rinsed with MilliQ water by hand two times using a syringe to remove any residual growth solution. During the flow step, the start of the growth time is recorded when the growth solution reaches the capillary, which can take 15–30 s depending on tubing length. During flow, the solution coming from the outlet was checked by visual inspection to confirm that it is clear immediately upon exiting the device, rather than red/blue, which would indicate significant secondary nucleation in the solution due to insufficient rinsing of the seeds from of the channel or other contamination. The final products appeared dark blue in color.

1.4. Gold nanostar characterization

Two different scanning electron microscopes were used for characterizing the morphology and uniformity for the AuNSTs grown on glass, PDMS, and ITO: ZEISS Supra 40VP SEM, 3-10 kV (California NanoSystems Institute) and FEI QUANTA 200 Field Emission Gun (Institute of Materials Science of Barcelona). The PDMS channels were removed from the glass or ITO base using a razor blade, then the substrates were prepared for electron microscopy characterization. With the first instrument (ZEISS Supra 40VP), the AuNSTs on glass and PDMS were coated with an Ir thin film (~3 nm) using an ion beam sputtering/etching system (South Bay Technology, Model IBS/e). The ITO substrates were imaged as-is without sputtering. Carbon tape was used to secure the sample to the sample holder along with copper tape to reduce charging. However, characterization for the non-conductive glass and PDMS was primarily performed with the second instrument (FEI QUANTA 200) due to its capability for environmental SEM, operating between 5 and 15 kV under low vacuum (60 Pa).

2. Details for Polydimethylsiloxane Channel Binding Process to Indium Tin Oxide

The use of an ITO substrate was essential for fast electron microscopy characterization of the fabricated nanostructures, without requiring environmental SEM. Although the PDMS channels readily bind to air or oxygen-plasma-treated glass *via* a condensation reaction, we observed that PDMS could not be strongly bound to ITO with this same method. Therefore, in order to facilitate binding of the PDMS channel with ITO, we first functionalized the ITO-coated glass with APTES as an adhesion layer. The air plasma treatment of the APTES-functionalized ITO and the PDMS channel resulted in the activation of both surfaces so that strong binding and

a liquid-tight seal could be achieved (**Figure S1**). The control experiments presented in **Figure S1** show that devices begin to leak within 5 min after flowing solutions with different viscosities between 150 and 500 μL/min in channels that did not have both an APTES coating and air plasma treatment. In **Figure S1C-E**, the strength of the PDMS-ITO binding can be further appreciated, where the subsequent removal of the PDMS channel leads to removal of the ITO coating from the underlying glass substrate. This effect is observed when the complete binding protocol is followed. In order to attach the gold seeds and perform *in situ* AuNST overgrowth, as described in the previous sections, the channels need to be re-functionalized with APTES. We show that the formation of dense AuNST layers on the ITO was achieved (see Main Text).

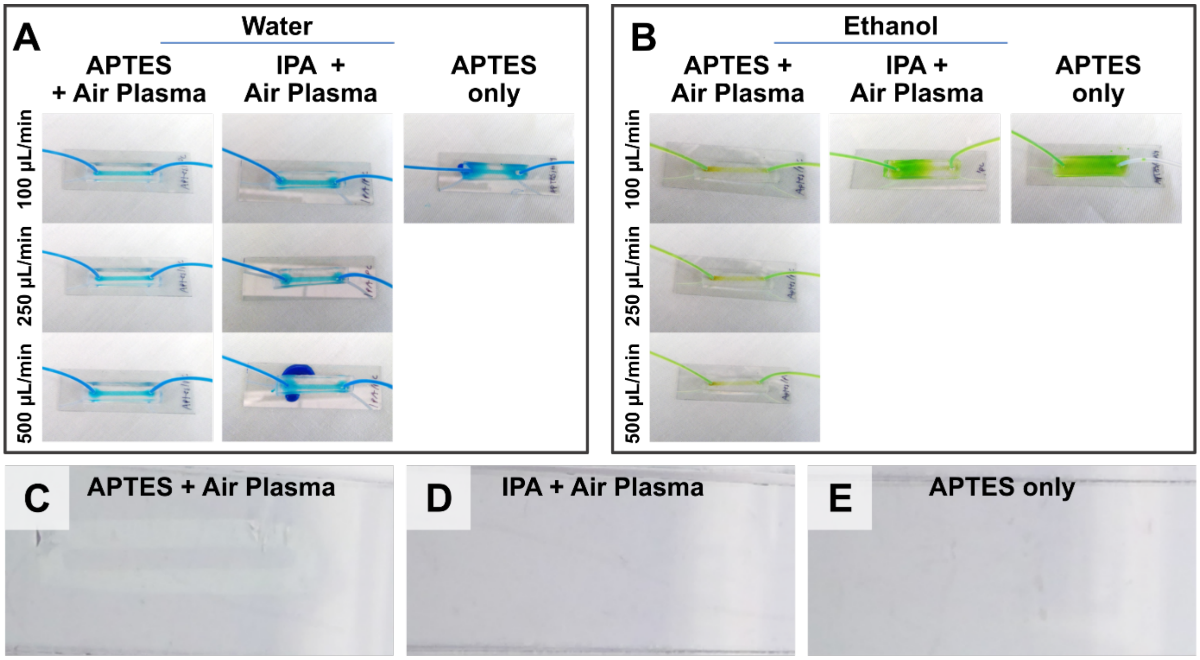

Figure S1. A: Digital photographs of polydimethylsiloxane (PDMS) channels bound to indium tin oxide (ITO) substrates under different conditions after flowing (**A**) water with (blue food coloring for contrast) and (**B**) ethanol (with green food coloring for contrast)**.** *Left to right:* after (3-aminopropyl)triethoxysilane (APTES) functionalization and air plasma activation, after rinsing with isopropyl alcohol (IPA) and air plasma treatment, and after only the APTES functionalization. Each row represents a different rate of flow tested over a 5 min period. Absent photographs indicate conditions where device failure occurred within the first 30 s of testing. **C-E:** Digital photographs of the underlying ITO-coated substrate after removal of the channel assembled with different surface treatments.

Because PDMS is naturally hydrophobic, the assembled channels were exposed to air plasma treatment for 1 min to assist in wetting and the removal of trapped air bubbles, which could be especially problematic for the herringbone channels with 3D features (**Figure S2**).

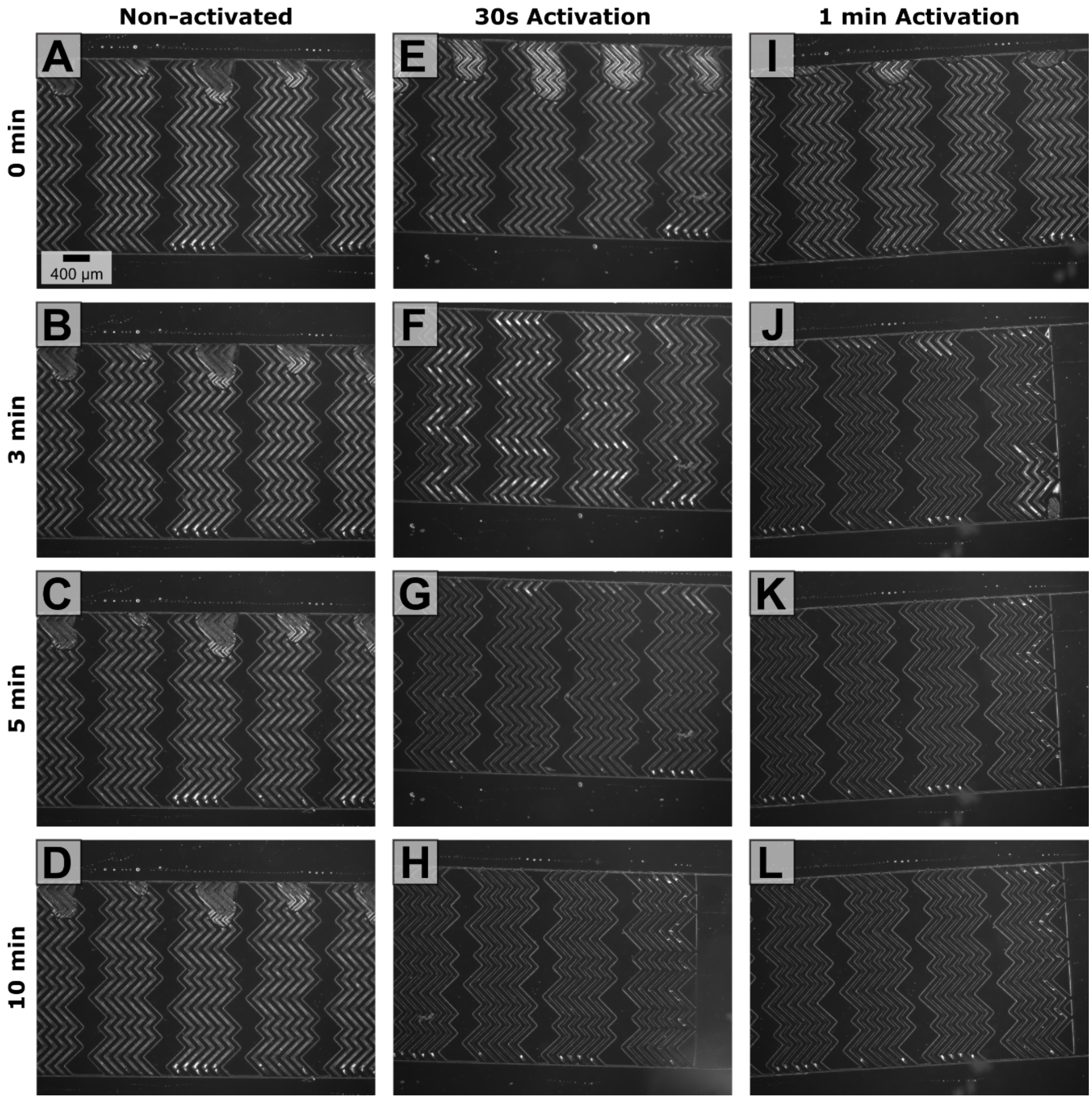

Figure S2. A-L: Optical microscope images of water flowing at 250 µL/min for the indicated time (*left*) through polydimethylsiloxane – indium tin oxide channels after (**A-D**) 0 s, (**E-H**) 30 s, and (**I-L**) 1 min air plasma activation.

When the second air plasma treatment is not performed, it is common to observe growth defects like those shown in **Figure S3.**

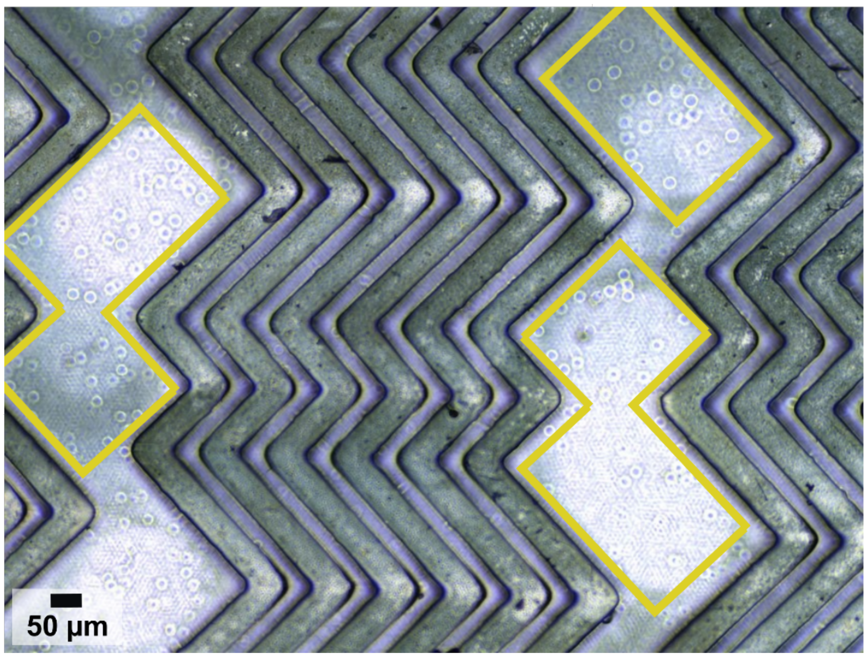

Figure S3. Brightfield microscope image showing growth defects caused by bubbles trapped in the gap regions (yellow outlined regions).

3. Environmental Scanning Electron Microscopy Characterization

With the optimized fabrication method and growth solution recipe described in the previous section, environmental SEM revealed branched structures on the PDMS channel and the ITO and glass substrates. In herringbone channels, AuNSTs were present at all areas within the 3D features, namely the "small herringbone," "large herringbone," and "gap" features indicated in **Figure S5**.

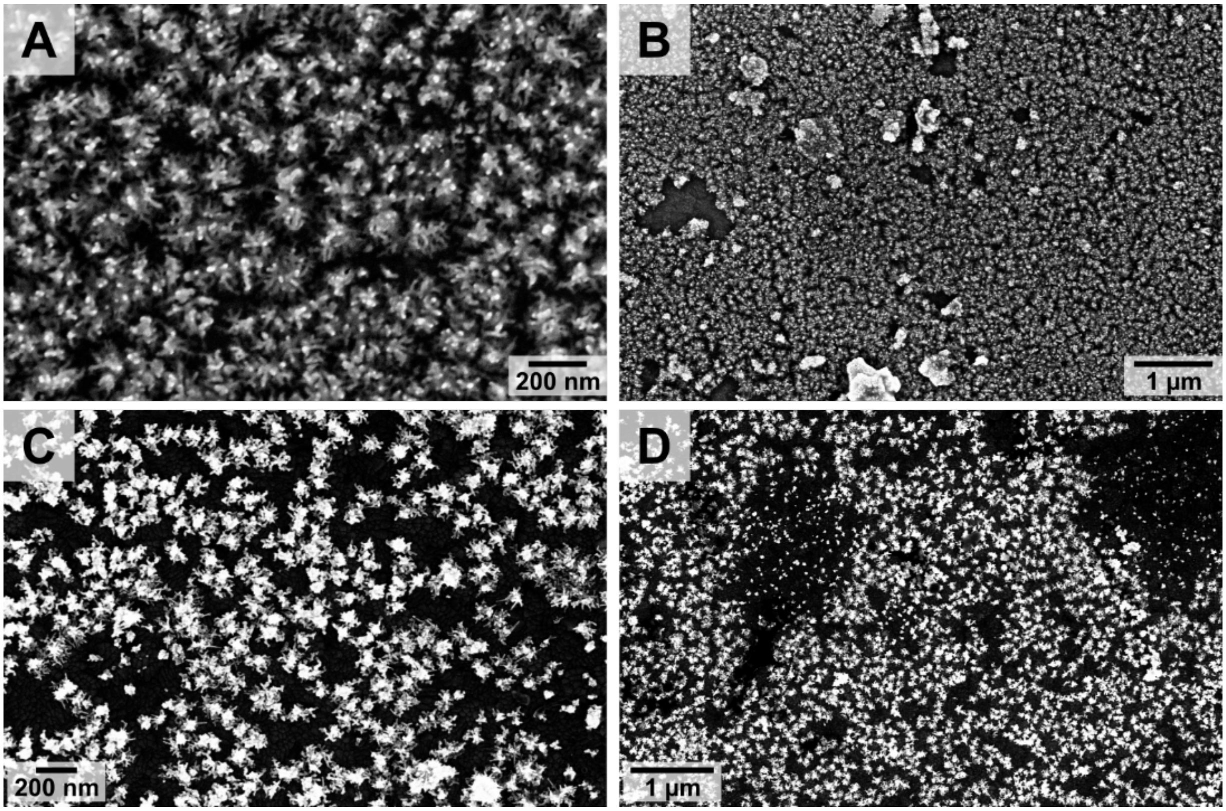

Figure S4. Scanning electron microscopy images of *in situ* grown gold nanostars stored for >1 month **A, B:** under vacuum at room temperature and **C, D:** immersed in aqueous solution.

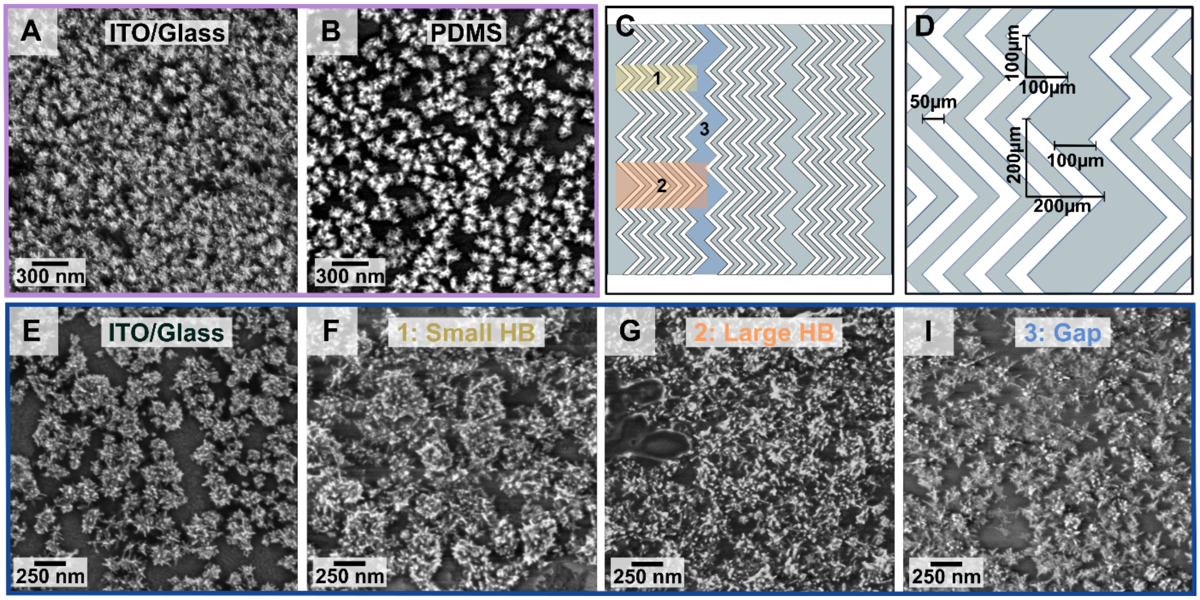

Figure S5. A, B: Scanning electron microscopy images of the branched products grown on (**A**) indium tin oxide (ITO)/glass and (**B**) polydimethylsiloxane (PDMS) in a featureless channel. **C, D:** Schematics showing different areas and dimensions of the herringbone PDMS channel. **E-H:** Scanning electron microscopy images of gold nanostars grown on the (**E**) substrate (ITO or glass), (**F**) the small herringbone (HB) feature (*area 1*), (**G**) the large HB feature (*area 2*) and (**H**) the gap between the HB repeats (*area 3*).

4. Characterization of Samples Growth with Fixed Growth Volume

In **Figure S6,** products grown using a fixed growth solution volume are shown.

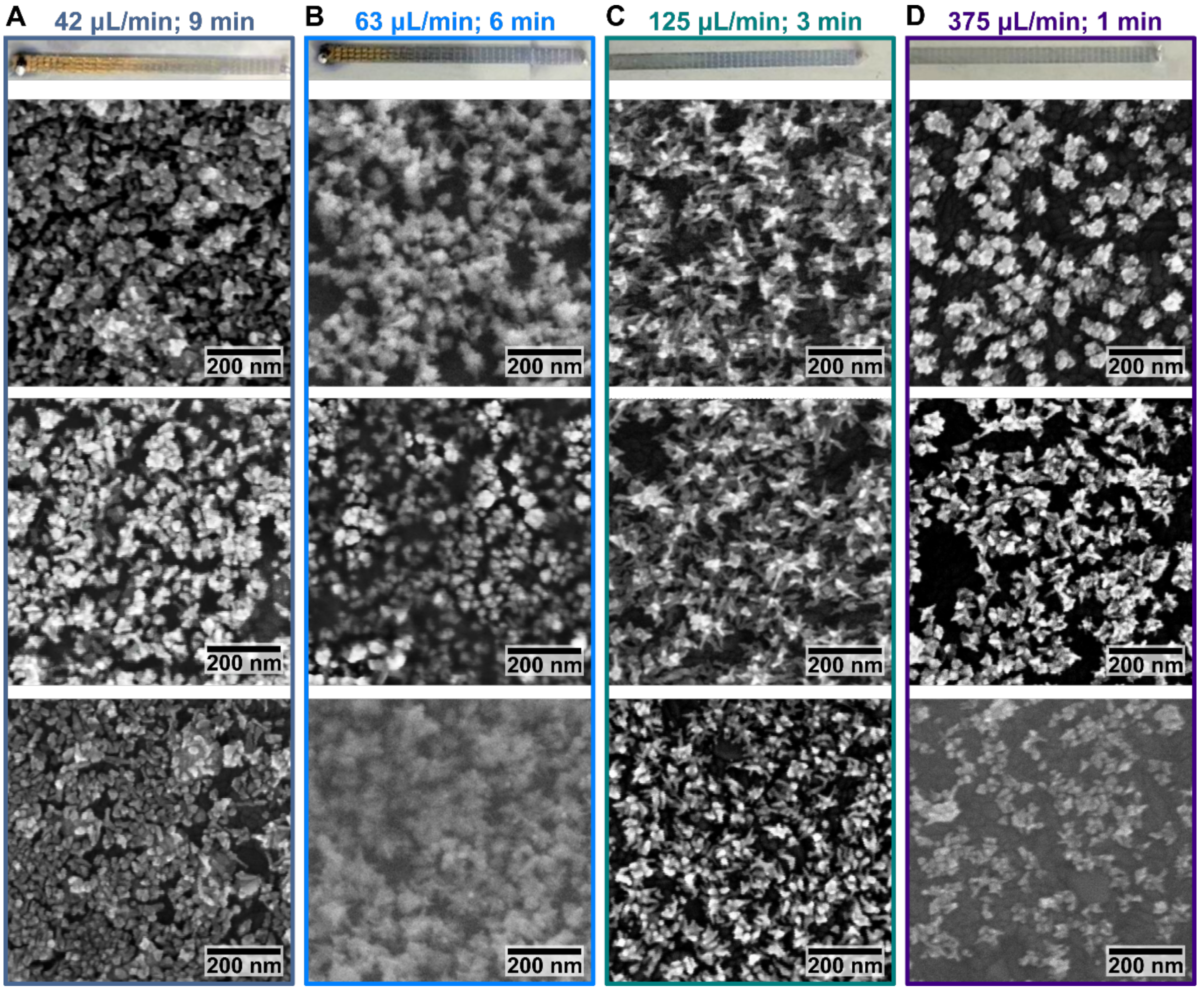

Figure S6. A-C: Digital photographs (*top*) and scanning electron microscopy images (*bottom*) of gold nanostars grown in flow in the herringbone channels using the same total growth solution volume with different flow rates and growth times.

5. Conventional Ultraviolet-Visible Spectroscopy Characterization

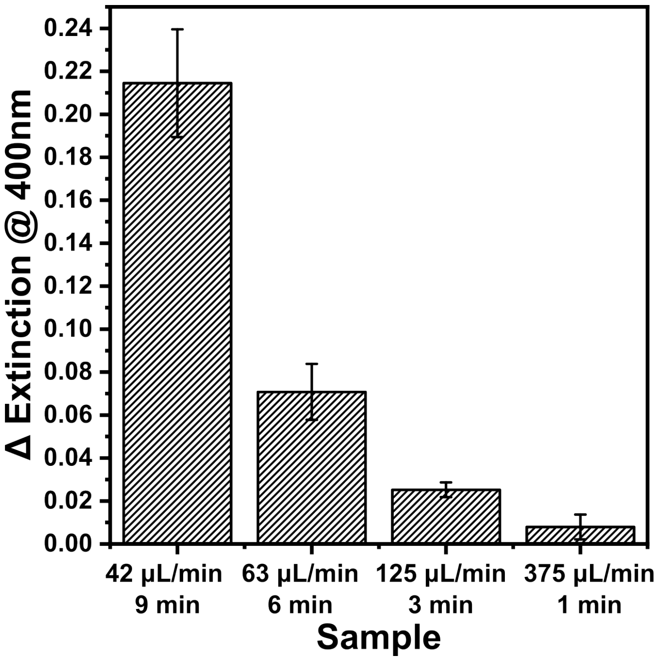

Figure S7. The difference in extinction at 400 nm between the inlet and the outlet regions of the channels grown at the conditions specified, showing the difference in amount of gold on the substrate at the distinct regions.

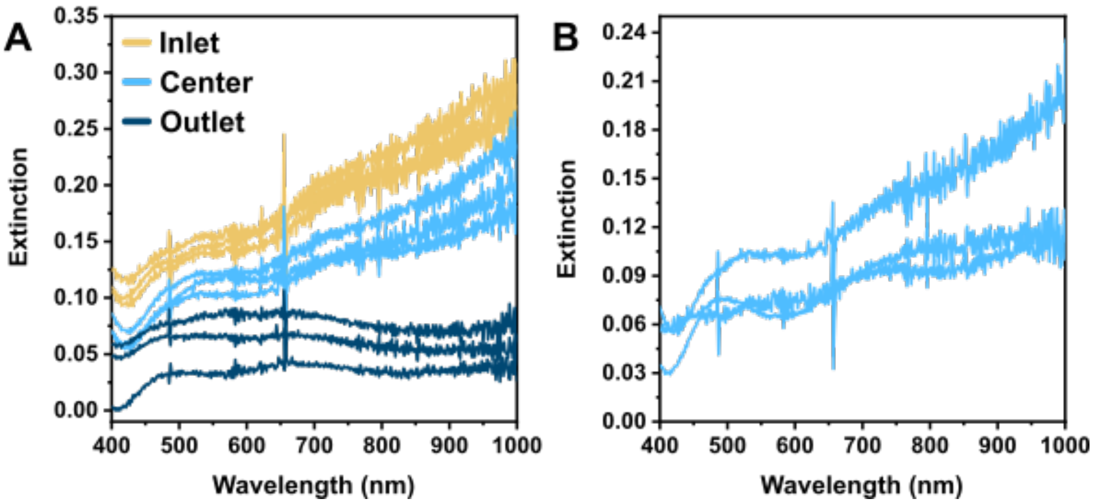

Figure S8. UV-visible spectroscopic characterization of channels grown at 125 µL/min for 3 min. **A:** Comparison of the spectra obtained at the inlet, center, and outlet of the channel, showing the gradient effect where the outlet has lower intensity. **B:** Repetitions of spectra obtained at the center of channels grown under the previously noted conditions.

6. MATLAB Evaluation of the Percentage of the Surface Covered with Gold

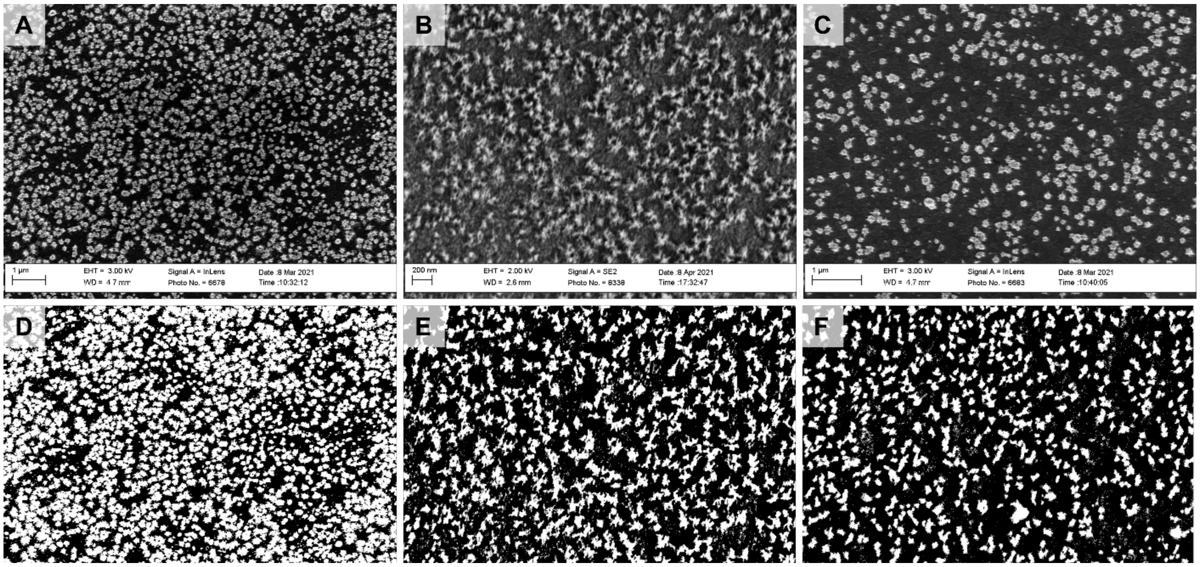

Figure S9. A-C: Originally captured scanning electron microscopy images of the (**A**) inlet, (**B**) center, and (**C**) outlet products synthesized in featureless channels. **D-F:** The corresponding black and white images created in MATLAB: cropped images were converted to black and white, binarized, then the percentage of white pixels was measured.

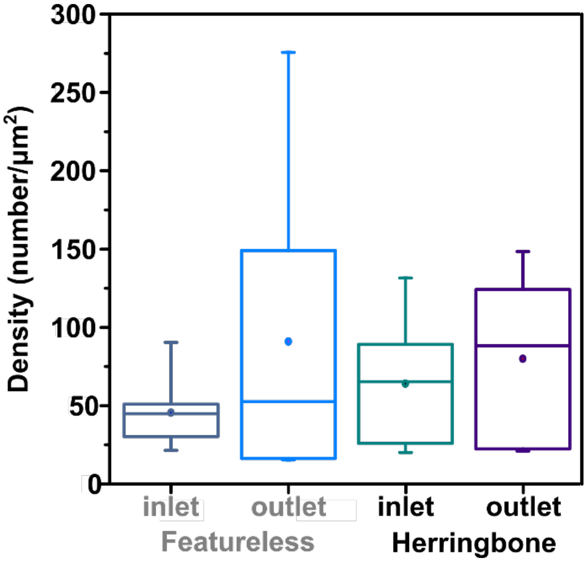

Figure S10. Measured density of nanostars (number of nanostars/μm^2) at the indicated regions of the different channel geometries.

7. Additional Data and Numerical Simulations

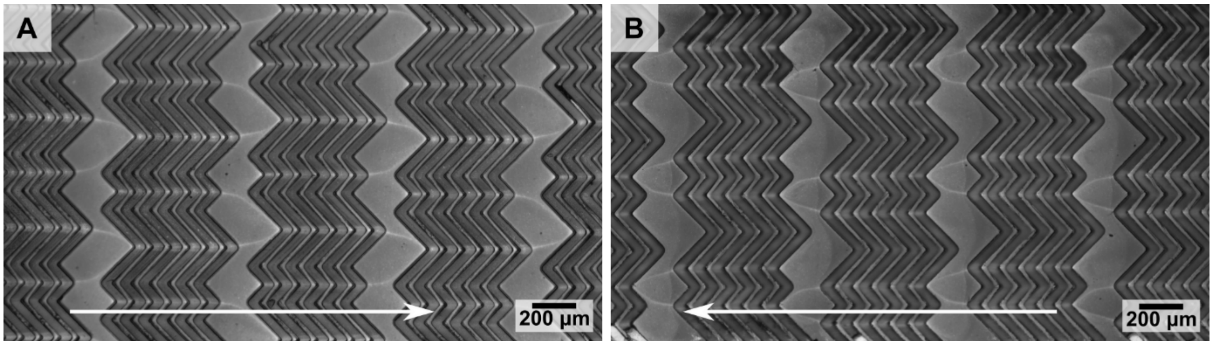

Figure S11. Additional brightfield images of the herringbone channels grown with different flow directions, indicated by the white arrows.

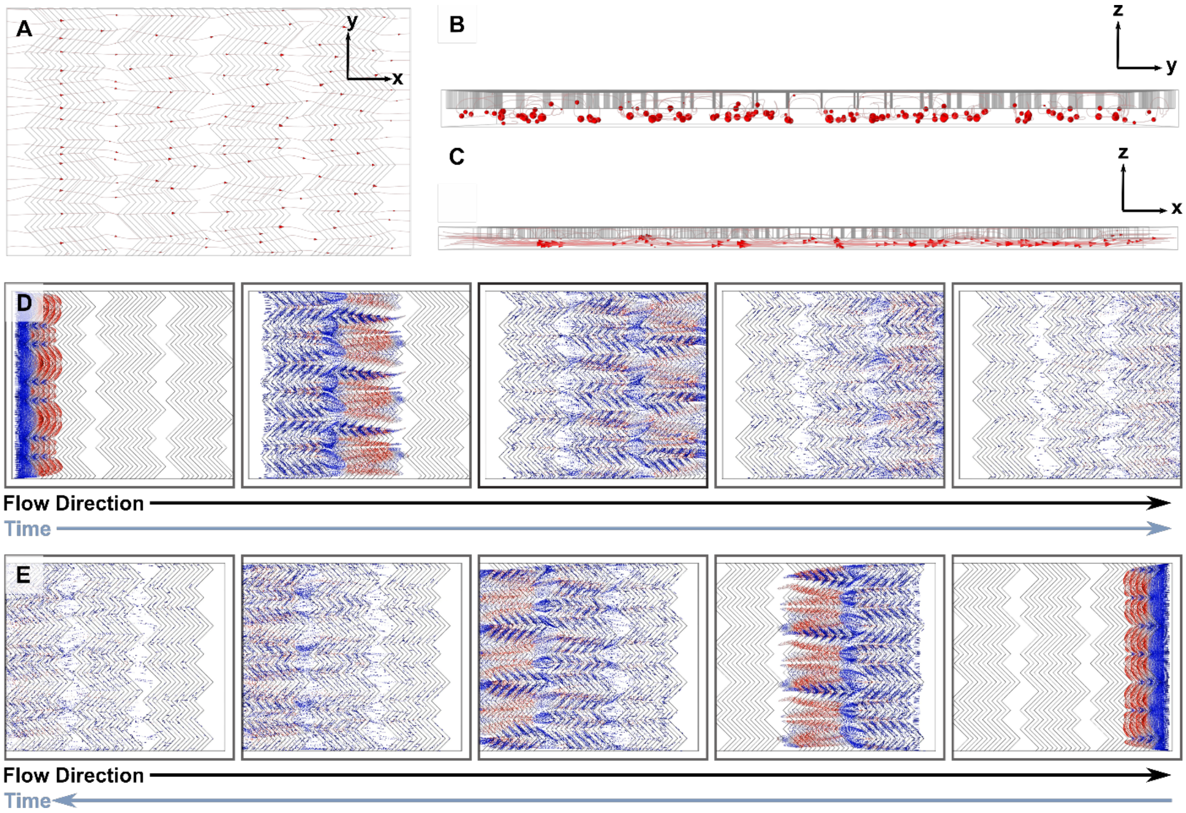

Figure S12. A-C: Velocity streamlines illustrating the velocity field in the herringbone channels. **D, E:** Snapshots at different time points from particle tracing simulations (**Videos S1,S2**) showing the flow patterns for different flow directions. Black arrow indicates flow direction and blue arrow indicates time progression.

Finite element analysis was used to simulate the velocity streamlines and flow velocities of contrast particles in a 3D computer aided design (CAD) model of the herringbone channel

geometry. The simulations were performed using the Laminar Flow (stationary study) and Particle Tracing for Fluid Flow (time-dependent study) modules of the COMSOL Multiphysics® software package. The Laminar Flow module was used to obtain the velocity and pressure distribution of water inside the device with an initial velocity of 0.00833 m/s at the inlets, matching the 100 μL/min flow condition. Then, a time-dependent study with the particle tracing module was used to visualize the flow profile (particle size: 10 nm, density: 2200 kg/m^3). Overall, 10,000 particles were released stepwise (0.1 s) over 0.5 s. Flow velocity streamlines showed vortex-like patterns within grooves of the herringbone channels (**Figure S12A-C**). The time lapse of the particle tracing shows that laterally across the channel that the particle flows are directed away from certain herringbone "peaks" depending on the applied flow direction (**Figure S12D, E**).

8. Additional Electron Microscopy Characterization of Gold Nanostars

Scanning electron microscopy images of the products obtained at different flow rates in the featureless and herringbone channels are shown in **Figures S13** and **S14**, respectively. For featureless channels, nanostructure formation is observed up to 1 mL/min due to tendency for device failure at flow rates higher than 1 mL/min. For herringbone channels, the upper limit is 250 μL/min because at this point, the surfaces start to present nearly complete coverage with gold.

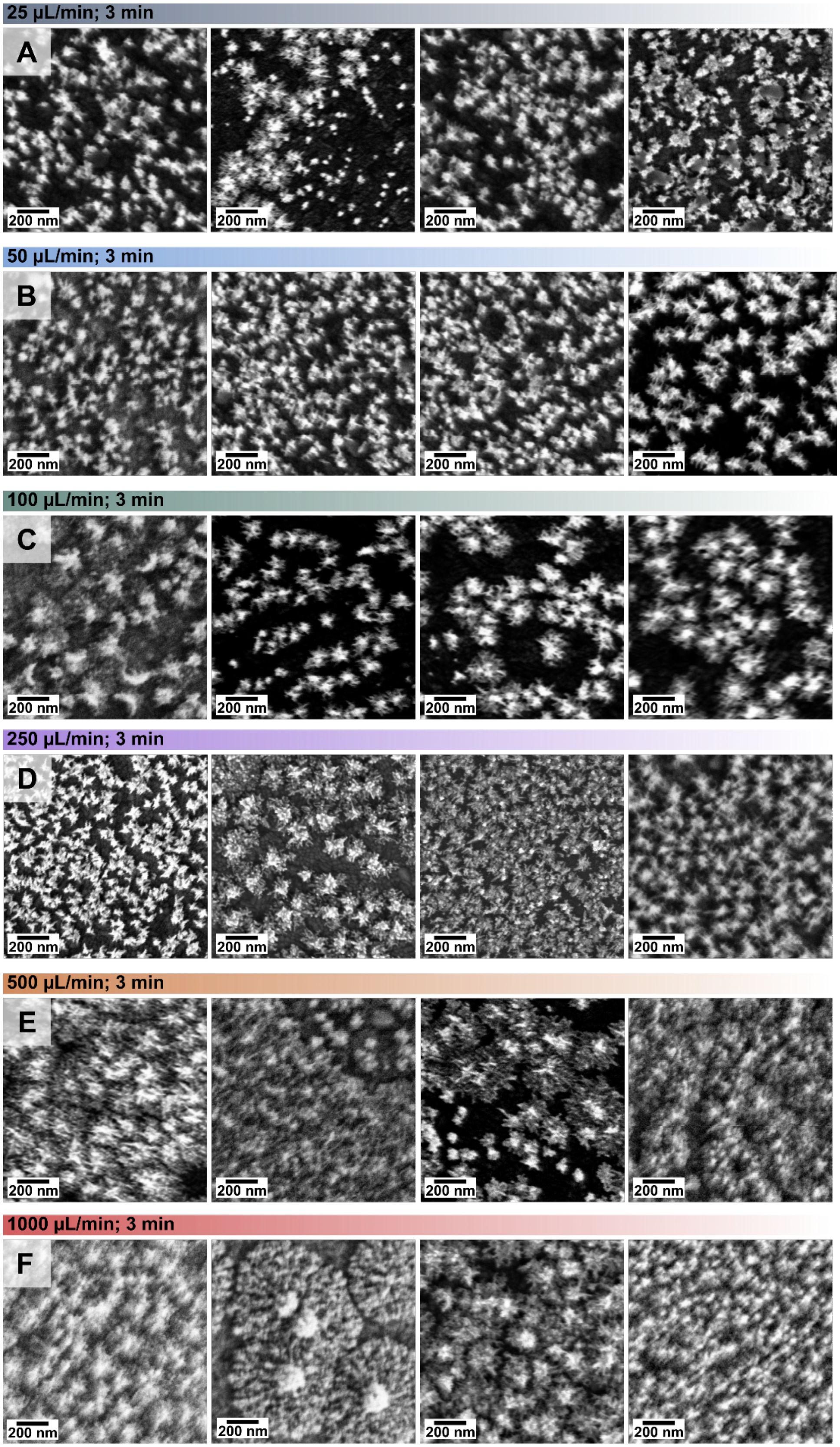

25 µL/min; 3 min
A
200 nm
50 µL/min; 3 min
B
200 nm
100 µL/min; 3 min
C
200 nm
250 µL/min; 3 min
D
200 nm
500 µL/min; 3 min
E
200 nm
1000 µL/min; 3 min
F
200 nm

Figure S13. Scanning electron microscopy images of branched products on indium tin oxide substrates after flowing growth solution for 3 min at the indicated flow rate in devices with featureless channels. Each image in the row for each tested flow rate corresponds to a separate sample. Excessive growth into films is increasingly observed at 500 µL/min and 1 mL/min, and the device begins to fail at higher flow rates.

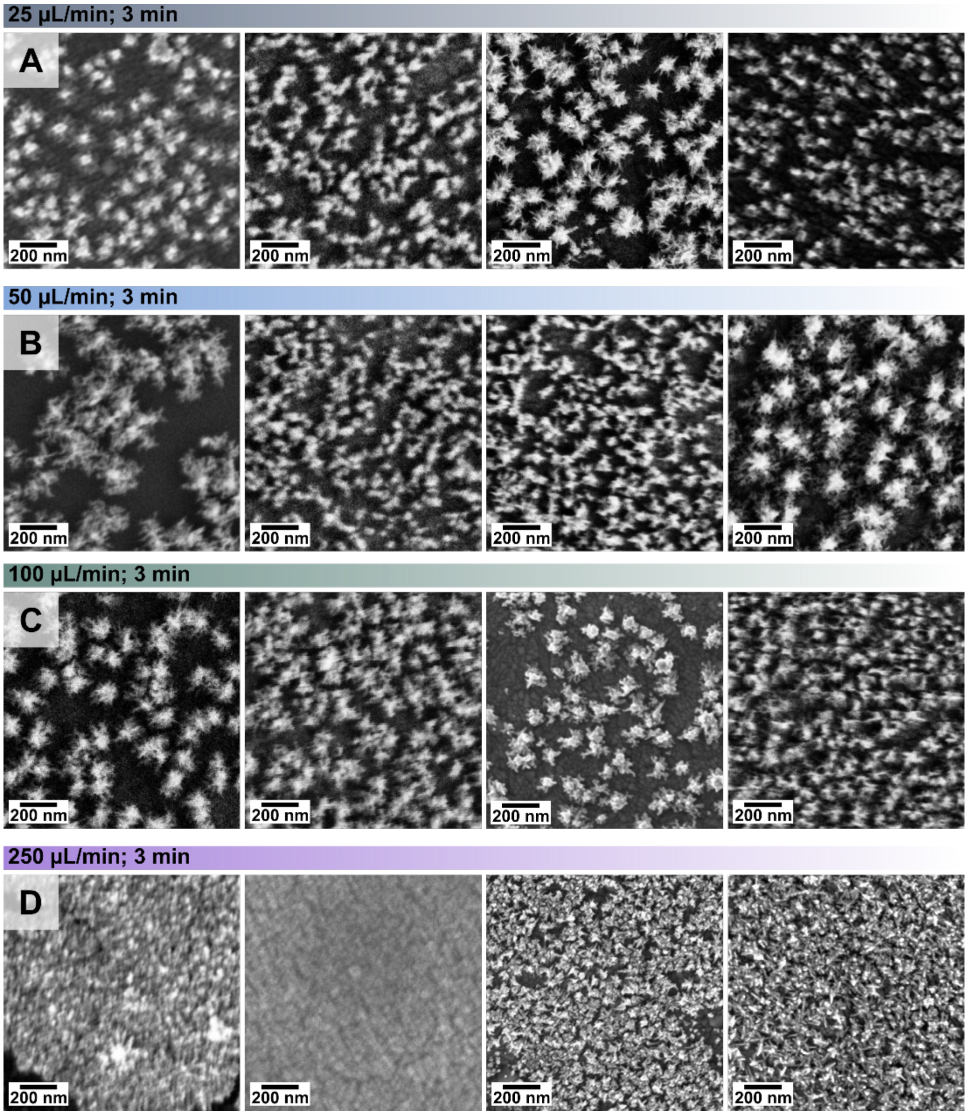

Figure S14. Scanning electron microscopy images of branched products on indium tin oxide substrates after flowing growth solution for 3 min at the indicated flow rate in herringbone (HB) channels. Each image in the row for each tested flow rate corresponds to a separate sample. For the HB channels, flow rates above 250 µL/min are not shown because this is the point at which the appearance of gold films dominates.

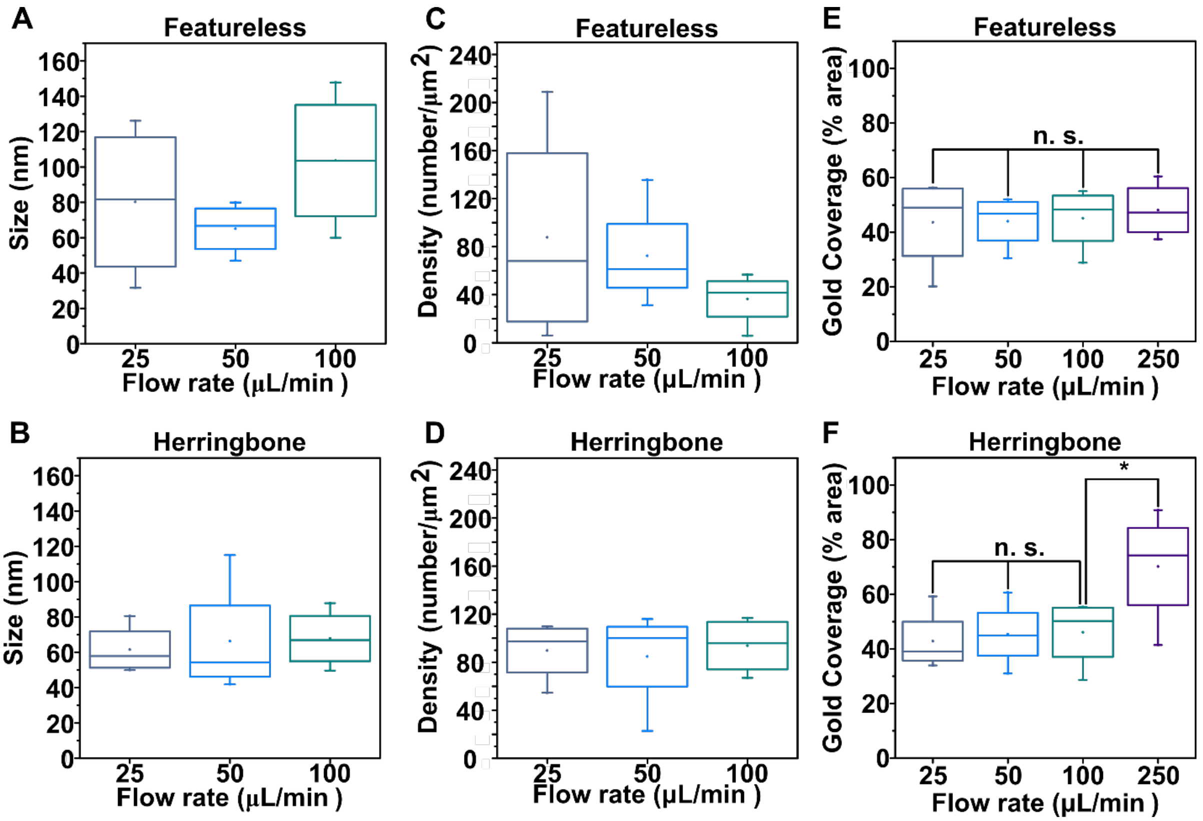

Figure S15. A-F: Box graphs of nanostar (**A,B**) size, (**C,D**) density, and (**E,F**) overall percent surface coverage of gold measured for featureless and herringbone devices.

The reduction of gold precursor in the outlet solution is complete when the measured extinction at 400 nm remains constant, corresponding to the onset of interband transitions of metallic gold. Based on our measurements, the gold solutions are sufficiently aged for the performance of our spectroscopic estimation of gold atom concentration after 2 h.

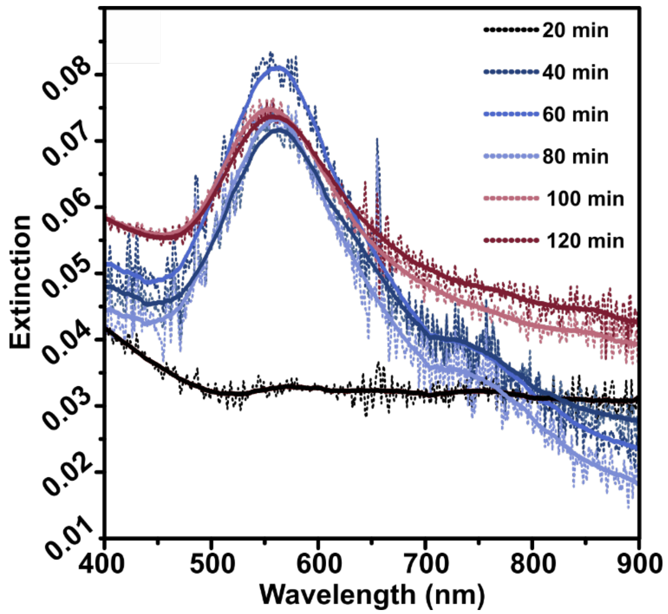

Figure S16. UV-visible spectra of the growth solution collected at the outlet aged from 20-120 min to evaluate the quantity of left over gold following the microfluidic synthesis.

The dense coverage of AuNSTs on the surface was observed at lower magnification, as well. From previous work, the consistency of the coverage and density at larger scales is determined by the uniformity and yield of the APTES and seeding steps (**Figures S17** and **S18**).[S1]

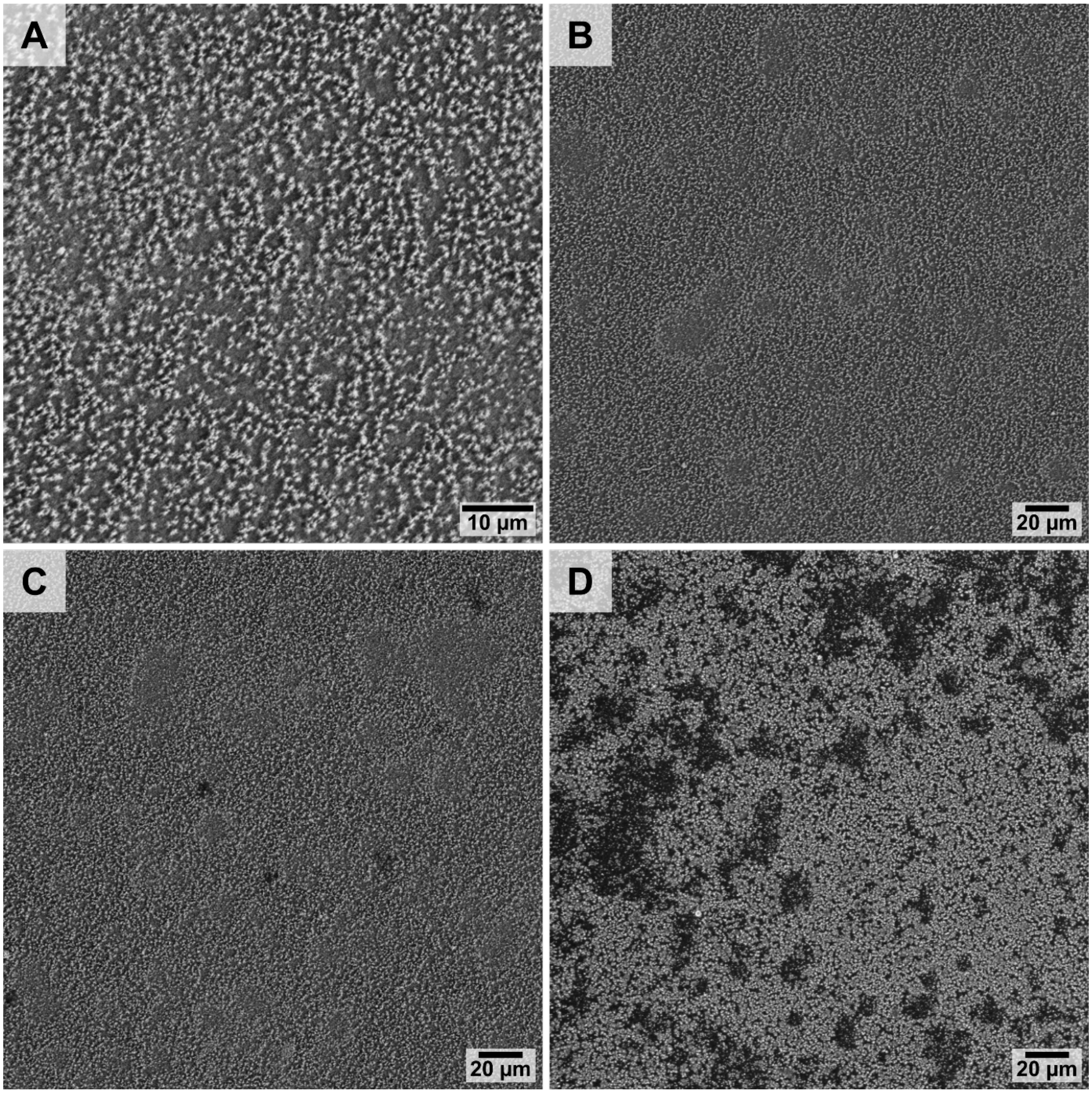

Figure S17. Additional low-magnification scanning electron microscopy images of gold nanostars synthesized in microfluidic devices with featureless channels.

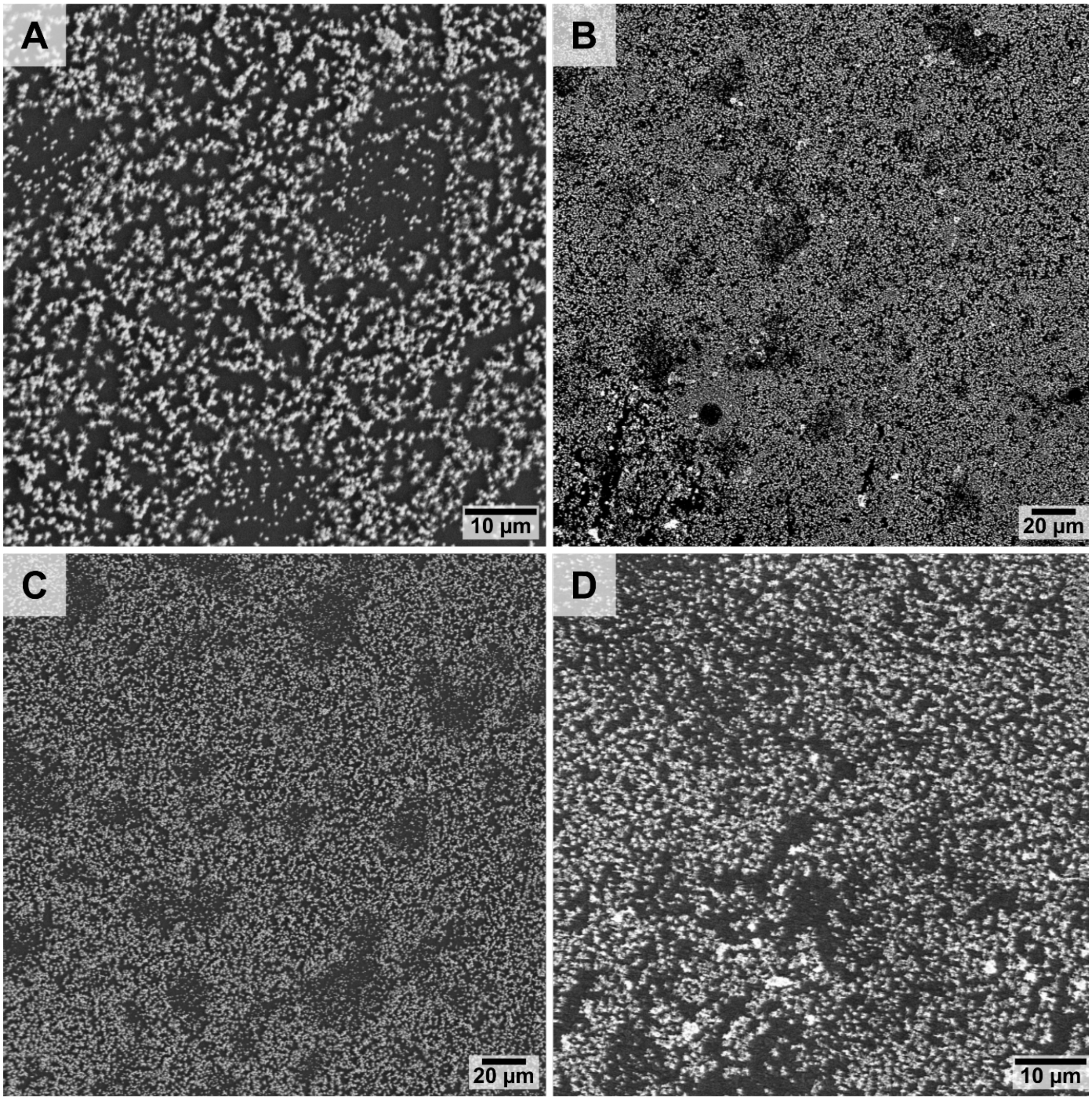

Figure S18. Additional low-magnification scanning electron microscopy images of gold nanostars synthesized in microfluidic devices with herringbone channels.

REFERENCES

(S1) Vinnacombe-Willson, G. A.; Chiang, N.; Scarabelli, L.; Hu, Y.; Heidenreich, L. K.; Li, X.; Gong, Y.; Inouye, D. T.; Fisher, T. S.; Weiss, P. S.; Jonas, S. J. *In Situ* Shape Control of Thermoplasmonic Gold Nanostars on Oxide Substrates for Hyperthermia-Mediated Cell Detachment. *ACS Cent. Sci.* **2020**, *6*, 2105–2116. https://doi.org/10.1021/acscentsci.0c01097.
